# Prevalence and factors influencing HIV testing behavior in adolescents and young adults: a systematic review and meta-analysis

**DOI:** 10.1016/j.pmedr.2025.103211

**Published:** 2025-08-12

**Authors:** Qiongge Yu, Hui Zhao, Dan Sun, Yuyan Liu, Yufeng Yu

**Affiliations:** School of Nursing, Chengdu University of Traditional Chinese Medicine, Chengdu, Sichuan Province 610075, China

**Keywords:** Adolescents, Young people, HIV testing, AIDS, Influencing factors

## Abstract

**Objective:**

To systematically evaluate the prevalence and influencing factors of human immunodeficiency virus (HIV) testing among adolescents and young adults (10–25 years old), providing a reference for optimizing testing strategies and supporting the global goal of eliminating HIV as a public health threat.

**Methods:**

A systematic review and meta-analysis were conducted following PRISMA (Preferred Reporting Items for Systematic Reviews and Meta-Analyses) recommendations. To assess the prevalence and factors influencing HIV testing among adolescents and young adults, databases were searched up to April 19, 2025. Publications were selected based on the inclusion criteria, and data were analyzed using Stata software.

**Results:**

A total of 22 studies were included. The results of the meta-analysis revealed that the HIV testing rate among adolescents and young adults was 32 % (95 %CI:0.24,0.39). The influencing factors included age ≥ 20 years old, educational level in secondary and higher, female, marital status, Asian or Hispanic or Latino ethnicity, sexually active, having two or more sexual partners, a history of pregnancy or impregnating a partner, living in rural areas.

**Conclusion:**

The results of this study suggest that adolescents and young adults have lower rates of HIV testing. Targeted measures should be implemented to facilitate the promotion of testing.

## Highlights

• The incidence of HIV testing among adolescents and young adults is low and needs to be further improved.
• A total of nine influencing factors for the testing were identified.
• Targeted measures should be implemented to facilitate the promotion of testing.

## 1 Introduction

Early detection and diagnosis of the human immunodeficiency virus (HIV) are essential for global acquired immunodeficiency syndrome (AIDS) prevention and control. The United Nations has also updated the three “95” AIDS targets to four “95” targets by 2021, requiring that 95 % of people living with HIV be diagnosed and 95 % of those diagnosed receive antiretroviral treatment. These goals aim to eliminate AIDS as a public threat by 2030. However, a large number of HIV-infected people remain, especially in the adolescent and young adult population. The World Health Organization (WHO) estimates that 4.5 million people become infected with HIV and other sexually transmitted infections (STIs) each year (World Health Organization, 2025a). Globally, about 480,000 young people between the ages of 10–24 are infected with HIV, with 140,000 of them between the ages of 10–19 (World Health Organization, 2025b). HIV testing is an important tool for achieving the 2030 goals (United Nations, 2025) and represents a critical step in AIDS prevention and treatment. The WHO has recommended HIV self-testing as part of pre-exposure prophylaxis (World Health Organization, 2025c), but testing behaviors vary significantly, particularly among adolescents and young adults. This demographic remains the least likely to be tested for HIV and to know their HIV status (Ajayi et al., 2020; Mwaba et al., 2020). Moreover, the mechanisms influencing HIV testing behavior are complex and are influenced by individual-level, societal-level, and outcome factors, such as varying policies and facilities for HIV testing between regions and informed consent requirements from parents and caregivers. These factors may discourage testing, especially for young people who are at a higher risk of HIV infection, thereby missing opportunities for early diagnosis and care (Schnall et al., 2016). Furthermore, current studies are mostly limited to a single geographic region or population, and significant heterogeneity is observed in the conclusions of different studies on the factors influencing HIV testing behavior. In recent years, high-income countries have significantly increased testing rates by providing access to self-service testing and community outreach, whereas low- and middle-income countries still rely on traditional health care facilities, with a relatively low testing coverage.

In this context, this study conducted a meta-analysis and systematic evaluation of the current status of HIV testing behaviors and influencing factors among adolescents and young adults. The results provide an evidence-based foundation for the development of precise HIV testing strategies, facilitating AIDS prevention and the identification of treatment gaps and social harms. This study is aligned with the global goal of HIV prevention and treatment.

## 2 Methods

### 2.1 Protocol and ethics review

This study was conducted in compliance with the PRISMA (Preferred Reporting Items for Systematic Reviews and Meta-Analyses) statement requirements and has been registered with Prospero (registration number CRD420251035569).

This study is a secondary research based on publicly published literature. All included data are derived from publicly available academic papers, with no use of unpublished raw data. The study did not involve direct contact with human/animal subjects, and personal information was not extracted. Therefore, further ethical approval was not required for this study.

### 2.2 Search strategy

Relevant articles were searched on PubMed, Web of Science, The Cochrane Library, Embase, China National Knowledge Infrastructure, Wanfang Database, Weipu Database, and China Biomedical Literature Database from the time of database inception to April 19, 2025. Studies related to the current status of HIV testing behavior and factors influencing HIV testing among adolescents and young adults were searched using a combination of subject and free word search terms. The Chinese search terms were “adolescents”, “youth”, “students”, “HIV”, “AIDS”, “testing behavior”, and “influencing factors”. The English search terms included “Adolescent”, “Youth”, “Student”, “HIV”, “AIDS”, “testing behavior”, and “influencing factors”. To ensure a comprehensive literature search, key phrases, entry terms, and medical subject headings terms were combined with Boolean operators (online Supplementary file Table A).

### 2.3 Study selection

Inclusion criteria: (1) The study population was a group of adolescents or young adults aged 10–25 years old; (2) The study investigated the current status of HIV testing behaviors and influencing factors among adolescents or young adults, reporting the Odds Ratio (OR) values and 95 % CI (confidence interval, CI) of the influencing factors; (3) The type of study was a cross-sectional study; (4) The original literature was in Chinese or English. Exclusion criteria: (1) Full-text articles were not available; (2) Reviews, systematic evaluations, and conferences. (3) Studies that were repetitively published, reused data, or had no available data.

### 2.4 Screening and data extraction

The results of the individual database searches were imported into EndNote software. The articles were screened, and data were extracted independently by two researchers. The following information was extracted from the literature: authors, publication date, country of study, total sample size, number of tests, and impact factors. Discrepancies between the two researchers were settled by a joint decision with a third researcher. In order to comprehensively extract and analyze the data on the influencing factors, the variables were recoded (online Supplementary file Table B).

### 2.5 Quality assessment

Quality assessment of the included literature was conducted independently by two investigators based on the quality assessment criteria recommended by the Agency for Healthcare Research and Quality (Chou et al., 2018) for cross-sectional studies. A total study quality assessment score of ≤3 was categorized as low quality, 4–7 as moderate quality, and ≥ 8 as high quality. Similarly, disagreements in assessments were settled by joint decision with a third researcher.

### 2.6 Statistical analysis

The prevalence of HIV testing behaviors and their influencing factors among adolescents and young adults was analyzed and presented using percentages, ORs, and 95 % CIs as effect sizes. Heterogeneity among the included studies was determined by the heterogeneity test, which was performed using a fixed effects model if I^2^<50 % and P>0.10, and a random effects model if I^2^ ≥ 50 % or *P* ≤ 0.10. The study was analyzed using stepwise exclusion for sensitivity analysis and Egger's test to assess the publication bias of the included studies. Notably, P<0.05 was indicative of potential publication bias; in such case, correction for bias was performed using the ‘trim and fill’ method. Stata version 16 software was used to conduct the meta-analysis.

## 3 Results

### 3.1 Study screening results

**Fig. 1:**
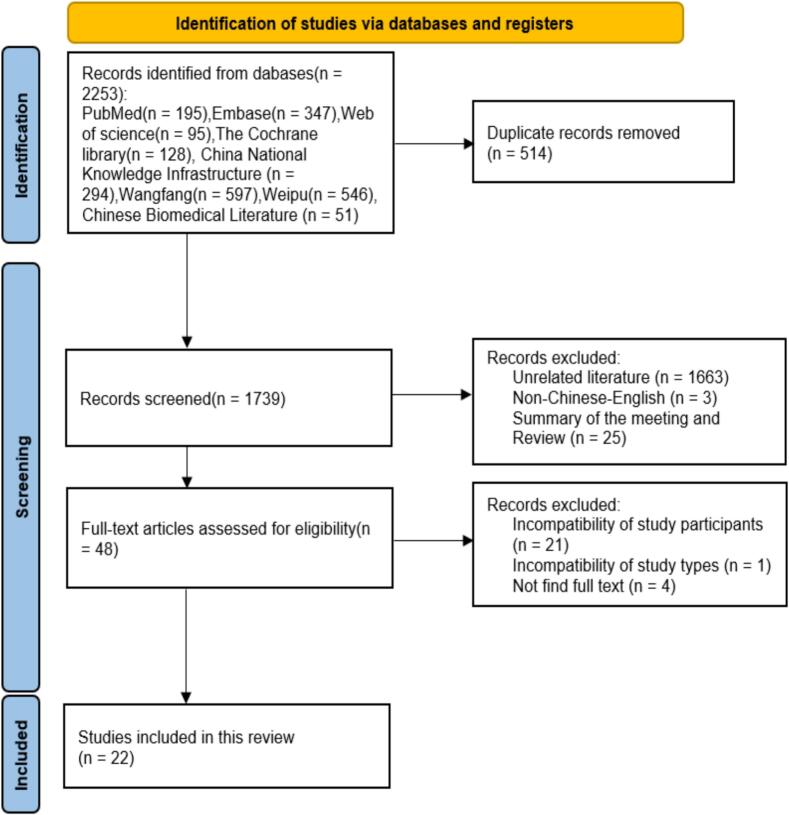
PRISMA flowchart for HIV testing rates and influencing factors in adolescents and young adults (inception to 2025).

A total of 2253 original documents were retrieved, and 514 duplicates were removed by importing them into EndNote. Following the primary screening by reading the titles and abstracts, 48 articles were included in the next step. Finally, the full texts of these articles were read, and 22 (Outlaw et al., 2022; Jawla et al., 2021; Maviso, 2024; Musumari et al., 2016; Ssebunya et al., 2018; Sonko et al., 2022; Ajiboye et al., 2023; Kowalska et al., 2024; Nshimirimana et al., 2022; Parchem and Molock, 2022; Leite et al., 2025; Sanga et al., 2015; Simelane et al., 2022; MacPhail et al., 2009; Cyrus et al., 2021; Williams Jr et al., 2025; Haney-Caron et al., 2021; Cheng et al., 2024; He et al., 2024; Shi et al., 2023; Zhen et al., 2021; Yang et al., 2017) documents were included in the final screening. The specific literature screening flowchart and results of this study are shown in Fig. 1.

### 3.2 Basic characteristics of included studies and quality assessment results

A total of 174,735 study participants were included across the 22 studies. Among them, one (Nshimirimana et al., 2022) article reported HIV testing rates and influencing factors for males and females separately; two (Ajiboye et al., 2023; MacPhail et al., 2009) studies reported influencing factors for males and females separately. Literature quality assessment scores ranged from 6 to 9, corresponding to medium to high quality. The basic characteristics and quality assessment of the included studies are shown in Table 1.

**Table 1:** Characteristics of included studies in the systematic evaluation of HIV testing rates among adolescents and young adults (inception to 2025).

Study	Country	Sample size	Number of tests	Influencing factors	Quality scores
Outlaw2022	The United States	105,147	17,265	Sexual partner, race, age, drinking, drug use	7
Jawla2021	The United States	2320	472	Race, age, age at first sex, number of sex partners, gender, grade, use alcohol/drug before last sexual intercourse, condom use, not using contraception at last intercourse, used contraceptive pills, considered suicide, obesity, ever smoked cigarettes, physical activity, described grades in school, hours spent watching television daily	8
Maviso2024	New Guinea	1275	222	Age, age at first sex, number of sex partners, condom use, marital status, education level, employment, residence, region, read newspaper/magazine, listen to the radio, watch television, internet/media access, own a mobile phone, knowledge of AIDS or HIV, ever paid for sex, sexually transmitted infection	8
Musumari2016	Thailand	519	95	Age, age at first sex, gender, condom use, sexually transmitted infection,income-producing, living status, currently having boy/girlfriend, HIV risk perception, sexually transmitted infections risk perception, ever pregnant or made someone pregnant, fear of HIV test result	9
Ssebunya2018	Uganda	1439	1117	Race, drug use, gender, education level, knowledge of AIDS or HIV, ever had sex	8
Sonko2022	Gambia	6194	1404	Race, age, age at first sex, sex, condom use, marital status, education level, residence, knowledge of AIDS or HIV, sexually transmitted infection, wealth index, have multiple sexual partners	7
Ajiboye2023	Ethiopia	7508	1674	Age, marital status, education level, employment, urban area size,non-spousal sexual partners in the last 12 months, circumcised, whether the last pregnancy had prenatal care	6
Kowalska2024	Poland	545	114	Not reported	6
Nshimirimana2022-female	Burundi	7218	1937	Age, number of sex partners, marital status, education level, residence, region, internet/media access, own a mobile phone, knowledge of AIDS or HIV, wealth index, have multiple sexual partners, religion, have health insurance, frequency of using the internet last month, discriminatory attitudes, stigma	6
Nshimirimana2022-male	Burundi	2860	490	Age, number of sex partners, marital status, education level, residence, region, Internet/media access, own a mobile phone, knowledge of AIDS or HIV, wealth index, have multiple sexual partners, religion, have health insurance, frequency of using the internet last month, discriminatory attitudes, stigma	6
Parchem2022	The United States	57	35	Not reported	6
Leite2025	Brazil	135	84	Not reported	6
Sanga2015	Tanzania	400	117	Age, gender, ever had sex, religion, forms of study, whether boarding, school ownership, has HIV been discussed with partner or parents, visited voluntary counseling and testing center	6
Simelane2022	Eswatini	1834	1227	Age, age at first sex, number of sex partners, gender, marital status, education level, employment, residence, ever had sex, wealth index, stigma	7
MacPhail2009	South Africa	7655	1945	Race, age, number of sex partners, condom use, marital status, education level, residence, ever pregnant or made someone pregnant, has HIV been discussed with partner or parents, sexual role in the last year, abnormal genital secretion, number of visits to clinics/health in the past 12 months	6
Cyrus2021	The United States	11,067	1142	Not reported	6
Williams2025	The United States	5732	4907	Race, age, age at first sex, gender, use alcohol/drug before last sexual intercourse, condom use, have multiple sexual partners, sexual identity	7
Haney-Caron2021	The United States	173	27	Not reported	6
Wu2024	China	4593	234	Age, gender, education level, residence, knowledge of AIDS or HIV, HIV risk perception, ever had sex, received school sex or AIDS education	7
He2024	China	2824	342	Sexual partner, age at first sex, number of sex partners, condom use, ways to find a sex partner or whether to find a sex partner online	6
Shi2023	China	4451	302	Gender, grade, residence, region, knowledge of AIDS or HIV, ever had sex, school ownership, school level, major	7
Dai2021	China	535	320	Age, drug use, sexually transmitted infection, knowledge of AIDS or HIV, received school sex or AIDS education, exposure to men who have sex with men social organizations in the last year, any homosexual anal sex in the last year, sexual role in the last year, temporary sex partners in the last year, gay stigma score or AIDS stigma score, knowledge of post-exposure prophylaxis	6
Yang2017	China	254	90	HIV risk perception, ways to find a sex partner or whether to find a sex partner online, any high-risk sexual behavior in the last month, willingness to test perimeter test, do you know any gay bars?	6

Note: Quality scores were derived from the quality assessment criteria recommended by the Agency for Healthcare Research and Quality. Studies with a score of ≤3 were classified as low quality, those with a score of 4 to 7 as moderate quality, and those with a score of ≥8 as high quality.

### 3.3 Meta-analysis results of the incidence of HIV testing behavior

**Fig. 2:**
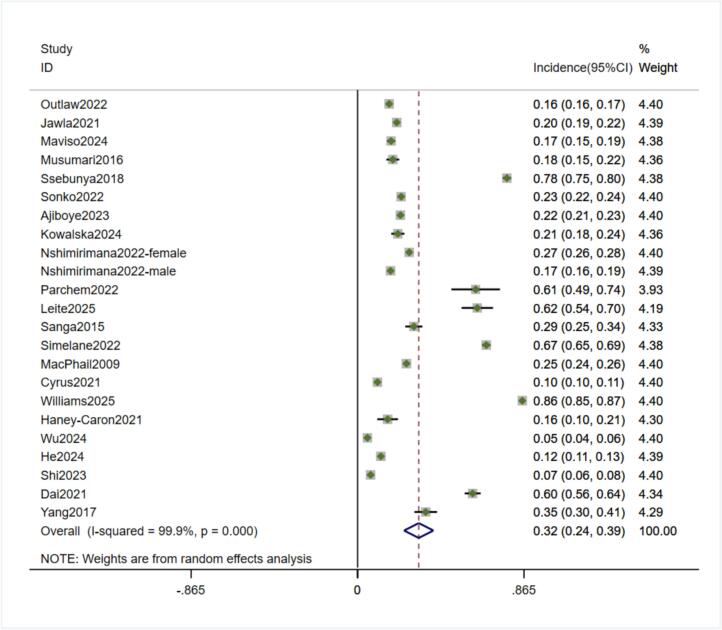
Forest plot of HIV testing incidence in adolescents and young adults (inception to 2025).

Significant heterogeneity was observed in the included literature (I^2^ = 99.9 %, P<0.00), so the analysis was performed using a random effects model. The results revealed that the prevalence of HIV-testing behaviors in the adolescent and young adult population was 32 % (95 %CI:0.24,0.39), as shown in Fig. 2.

### 3.4 Results of meta-analysis of factors influencing HIV testing behavior

A total of 17 influencing factors were identified. Among them, heterogeneity (I^2^>50 %) was observed in the following factors: female, age 15–19 years, secondary or higher education level, married, living in an urban area, poor and middle wealth index, black race, having been circumcised, having had sex, age of sexual debut <15 and ≥ 15 years, having same-gender sex, using a condom, having two or more sexual partners, and a history of pregnancy or impregnating a partner; hence, analysis was conducted using a random effects model. In contrast, no statistically significant heterogeneity (I^2^<50 %) was found in the following factors: age ≥ 20 years, no education or primary education level, being unmarried, residing in a rural area, rich and richest wealth index, Asian or Hispanic or Latino ethnicity, having a job, having same-gender and opposite-gender sex, a history of sexually transmitted infections, and drinking alcohol or using drugs before the last sexual intercourse; thus, these factors were analyzed using a fixed effects model. The results showed that the following factors were associated with a tendency for HIV testing: being female, ≥20 years of age, secondary and higher education level, being married, Asian or Hispanic or Latino ethnicity, being sexually active, having two or more sexual partners, and a history of pregnancy or impregnating a partner (P<0.05). Conversely, being unmarried and living in a rural area were barriers to HIV testing behavior (P<0.05). The specific results are shown in Table 2.

**Table 2:** Meta-analysis of influencing factors included in the systematic review of HIV testing rates among adolescents and young adults (inception to 2025).

Influencing factors	Studies(N)	Heterogeneous results		Meta-analysis results	
		I^2^(%)	P	OR	95 %CI
Gender					
Female	8	75.9	<0.00	1.45	(1.21,1.75)
Age					
15–19	4	95.1	<0.00	1.05	(0.48,2.29)
≥20	6	39.1	0.11	1.66	(1.45,1.90)
Education level					
No education	2	0.0	0.34	0.40	(0.13,1.21)
Primary	6	26.4	0.22	1.11	(0.93,1.32)
Secondary and higher	7	65.0	<0.00	1.41	(1.13,1.75)
Marital status					
Unmarried	2	10.6	0.29	0.66	(0.49,0.89)
Married	4	83.0	<0.00	2.10	(1.38,3.19)
Residence					
Urban	2	92.9	<0.00	1.25	(0.70,2.23)
Rural	4	34.9	0.19	0.82	(0.68,0.99)
Wealth index					
Poor	3	83.3	<0.00	1.23	(0.88,1.73)
Middle	2	82.0	<0.00	1.38	(0.88,2.16)
Rich	2	0.0	0.97	1.10	(0.93,1.31)
Richest	2	42.8	0.17	1.20	(0.92,1.57)
Race					
Black	4	97.3	<0.00	0.86	(0.29,2.59)
Hispanic or Latino	3	0.0	0.42	1.36	(1.16,1.59)
Asian	2	0.0	0.40	1.66	(1.15,2.40)
Employment					
Yes	2	0.0	0.51	1.02	(0.85,1.22)
Circumcised					
Yes	2	86.3	0.01	1.06	(0.39,2.88)
Ever had Sex					
Yes	5	78.9	<0.00	1.87	(1.31,2.66)
Age at first sexual intercourse					
<15	2	95.0	<0.00	1.93	(0.48,7.81)
≥15	6	96.2	<0.00	0.84	(0.37,1.95)
Sexual partner					
Homosexual	2	92.1	<0.00	1.85	(0.58,5.89)
Homosexual and heterosexual	2	24.5	0.25	1.05	(0.78,1.41)
Condom use					
Use	5	56.1	0.04	0.94	(0.77,1.13)
Number of sex partners					
≥2	6	59.3	0.02	1.75	(1.33,2.31)
Ever pregnant or made someone pregnant					
Yes	2	90.1	<0.00	2.32	(1.12,4.81)
Sexually transmitted infection					
Yes	3	9.8	0.33	0.94	(0.58,1.52)
Use alcohol/drug before last sexual intercourse					
Yes	2	1.7	0.31	1.02	(0.79,1.31)

Note: The wealth index was constructed using household assets (housing, savings, vehicles) and adjusted for liabilities. The wealth values were sorted from lowest to highest and divided equally into five groups: Poorest (bottom 20 %), Poor (21 %–40 %), Middle (41 %–60 %), Rich (61 %–80 %), and Richest (top 20 %) based on the distribution of index values in the study sample.

### 3.5 Sensitivity analysis

Sensitivity analyses of the incidence of HIV testing behaviors were performed using the leave-one-out method. The meta-analysis results showed no significant change after the exclusion of any individual article, indicating the stability of the results.

### 3.6 Publication bias

Publication bias was assessed using Egger's test, showing no publication bias (*P* = 0.13).

## 4 Discussion

The results of this study indicated that the prevalence of HIV testing behavior in the adolescent and young adult population was 32 %, which is lower than the studies of Myles (Myles et al., 2020) and Musonda (Musonda et al., 2023). This discrepancy may be attributed to the different regions where the study was conducted and the methodology of the study survey.

The present study showed higher HIV testing rates among females, aged ≥20 years, and married individuals, which is consistent with previous studies (Ajayi et al., 2020; Simelane et al., 2022; Iwelunmor et al., 2025; Segawa et al., 2022). In the younger age group, the prevalence of HIV testing behaviors increased with age, which could be due to young people being progressively more aware of sexual risk behaviors and being more conscious of their health status compared to adolescents. Moreover, the higher HIV testing rate among women compared to men may be related to women's greater awareness of health management and more access to medical services, such as preconception and prenatal or pre-delivery testing. In addition, married groups may be more concerned about the impact of disease infection on fertility, children, and family than unmarried groups.

The present study demonstrates higher uptake of HIV testing among Asian, Hispanic or Latino individuals compared to other ethnic groups. Adolescents and young adults with secondary and tertiary education are more likely to be tested for HIV compared to those with no education or only primary education, which is consistent with previous studies (Outlaw et al., 2022; Jawla et al., 2021; Musonda et al., 2023; Pachena and Musekiwa, 2022). With increasing levels of education, individuals may have a better understanding of HIV and a better assessment of their risk of infection. Moreover, studies have shown that both HIV knowledge and risk perception influence the acceptability of HIV testing (Ajayi et al., 2019). Therefore, educating adolescents and young adults about HIV and other STIs is not only conducive to HIV testing but also to improving their health status.

The present study shows that people who have had sex, have had more than two sexual partners, and have a history of pregnancy or impregnanting a partner are more likely to get tested for HIV, which is consistent with previous studies (Pachena and Musekiwa, 2022; Kawuki et al., 2023). Individuals with multiple sexual partners may feel at higher risk for HIV infection and be more prone to seek HIV prevention and testing services. The greater proportion of individuals with a history of pregnancy who are tested reflects the successful implementation of fertility-related HIV testing and the requirement in some areas for co-testing of pregnant partners. In addition, having a history of pregnancy, particularly when accompanied by evidence of recent unprotected sex and other risk factors, has been associated with an increased rate of HIV testing.

HIV testing rates are lower among people living in rural areas than in urban areas (Asingwire et al., 2025), which may be attributed to limited access to healthcare resources or stigma and avoidance due to sociocultural factors.

Furthermore, the surveyed countries included in the original research for this study are relatively decentralized. The source of heterogeneity may include differences in healthcare systems, testing policies, socio-economics, and cultural attitudes toward HIV testing across countries. Differences are observed in terms of socioeconomics among the countries included in the original literature. For example, the United States is a developed country, China is a developing country, and the analysis included other lower-middle and low-income countries such as Tanzania and Ethiopia. Moreover, significant differences in the level of healthcare services are observed across countries, such as gaps in HIV testing policies, free testing services, and the allowance of anonymous testing to reduce the impact of stigmatization on testing behaviors. Due to religious beliefs and traditional culture, some groups have discriminatory attitudes toward people living with HIV, deterring individuals from testing due to fear of discrimination.

Considering the geographic diversity of the included studies, appropriate measures should be tailored to increase HIV testing rates based on the country, especially in terms of resource allocation and program design. In low-income and middle-income countries, international assistance can be maintained or increased, and community crowdfunding can be carried out. Meanwhile, low-cost and wide-coverage strategies can be adopted, such as training community health workers to conduct testing, thereby reducing the cost of testing due to the lower salary costs. In high-income countries, testing and diagnostic technologies and personalized services can be developed to improve diagnostic accuracy, track high-risk communities through big data, increase the concentration of testing resources, improve health insurance coverage, and reduce testing costs.

At this stage, most studies on HIV testing rates have performed quantitative surveys. In the future, longitudinal designs, qualitative surveys, or intervention studies could be conducted to identify intrinsic drivers of HIV testing among adolescents and young adults to inform the selection of more optimal testing methods.

Nevertheless, the limitations of the present study should be acknowledged. (1) Potential selection bias may be attributed to some studies including only homeless and sexual minorities in younger age groups as respondents. In addition, most of the studies were conducted in the Americas and Africa, which may have biased the results due to regional differences and the unique HIV prevalence characteristics of these regions. (2) Only cross-sectional studies were included in this study. (3) Some of the influencing factors could not be integrated due to insufficient data, which may lead to inadequate analysis of the influencing factors. Additional studies are needed to explore the HIV testing perceptions and influencing factors among the younger population to seek a better and more feasible means or modality of testing.

## 5 Conclusion

The estimated prevalence of HIV testing behavior among adolescents and young adults is 32 % (95 % CI: 0.24,0.39), which is low and may affect the effectiveness of HIV prevention and control. In the present study, factors facilitating HIV testing included being aged ≥20 years, having a secondary or higher education, being female, being married, identifying as Asian, Hispanic or Latino, having had sexual intercourse, having more than two sexual partners, and having a history of pregnancy or impregnating someone. Conversely, being unmarried and residing in rural areas were found to deter HIV testing.

Thus, targeted strategies can be formulated to raise HIV testing rates, thereby contributing to the global goal of eliminating AIDS as a public health threat.

## CRediT authorship contribution statement

**Qiongge Yu:** Writing – original draft, Formal analysis, Data curation, Conceptualization. **Hui Zhao:** Data curation. **Dan Sun:** Data curation. **Yuyan Liu:** Formal analysis. **Yufeng Yu:** Writing – review & editing.

## Ethical approval and consent to participate

Not applicable.

## Funding statement

This research did not receive any specific grant from funding agencies in the public, commercial, or not-for-profit sectors.

## Declaration of competing interest

None.

## Data Availability

The datasets used and/or analyzed during the current study are available from the corresponding author upon reasonable request.

## Appendix A Supplementary data

Supplementary material Image 1
