# Supplementary material for: Prevalence and factors influencing HIV testing behavior in adolescents and young adults: a systematic review and meta-analysis
Source: Prev Med Rep. 2025 Aug 12;57:103211. doi: 10.1016/j.pmedr.2025.103211 (PMC12362408; doi:10.1016/j.pmedr.2025.103211)
Supplement: Supplementary file 1 — Supplementary material [file mmc1.docx]

Table A Details of the literature search strategy in the systematic evaluation of HIV testing rates among adolescents and young adults

| Search Number | Query |
| --- | --- |
| #1 | "Youth"[Mesh] |
| #2 | ((((Youth adult[Title/Abstract]) OR (young[Title/Abstract])) OR (Adolescent[Title/Abstract])) OR (teen[Title/Abstract])) OR (student[Title/Abstract]) |
| #3 | #1 OR #2 |
| #4 | "HIV"[Mesh] |
| #5 | ((AIDS Virus[Title/Abstract]) OR (Human Immunodeficiency Virus[Title/Abstract])) OR (AIDS[Title/Abstract]) |
| #6 | #4 OR #5 |
| #7 | (((test[Title/Abstract]) OR (testing behavior[Title/Abstract])) OR (self-test[Title/Abstract])) OR (self testing[Title/Abstract]) |
| #8 | #3 AND #6 AND #7 |

Table B Impact factors recoding details of included studies in the systematic evaluation of HIV testing rates among adolescents and young adults

| Influencing factors | Recoded groupings | Influencing factors | Recoded groupings |
| --- | --- | --- | --- |
| Age | ≤14 | Sexual partner | Heterosexual |
|  | 15~19 |  | Homosexual |
|  | ≥20 |  | Homosexual and heterosexual |
| Gender | Male | Marital status | Unmarried |
|  | Female |  | Married |
| Education level | No education | Residence | Urban |
|  | Primary |  | Rural |
|  | Secondary and higher |  |  |
| Race | Black | Wealth index | Poor |
|  | Asian |  | Middle |
|  | Spanish or latin |  | Rich |
|  | Others |  | Richest |
| Employment | Yes | Circumcised | Yes |
|  | No |  | No |
| Ever had Sex | Yes | Age at first sexual intercourse | ＜15 |
|  | No |  | ≥15 |
| Number of sex partners | 1 | Ever pregnant or made someone pregnant | Yes |
|  | ≥2 |  | No |
| Sexually transmitted infection | Yes | Use alcohol/drug before last sexual intercourse | Yes |
|  | No |  | No |
